# Serum ANCA and Overall Mortality: A 10-Year Retrospective Cohort Study on 1,024 Italian Subjects

**DOI:** 10.3389/fimmu.2021.714174

**Published:** 2021-09-10

**Authors:** Enrico Brunetta, Giacomo Ramponi, Marco Folci, Maria De Santis, Emanuela Morenghi, Elena Vanni, Elena Bredi, Raffaello Furlan, Claudio Angelini, Carlo Selmi

**Affiliations:** ^1^IRCCS Humanitas Research Hospital, Rozzano, Italy; ^2^Department of Biomedical Sciences, Humanitas University, Pieve Emanuele, Italy; ^3^Unit of Rheumatology and Clinical Immunology, IRCCS Humanitas Research Hospital, Rozzano, Italy

**Keywords:** ANCA, retrospective, cohort, vasculitis, rheumatoid arthritis

## Abstract

**Background:**

Antineutrophil cytoplasmic antibodies (ANCA) are primarily involved in the pathogenesis of ANCA-associated vasculitides (AAV). However, ANCA may also be present in healthy subjects and in patients with autoimmune disorders different from AAV. We hypothesized that serum ANCA are associated with a worse prognosis in disorders other than AAV.

**Objective:**

We investigated the association between the overall survival and the presence of serum ANCA in 1,024 Italian subjects with various testing indications in a 10-year interval.

**Methods:**

In this retrospective cohort study, a population of 6,285 patients (many of whom were subsequently excluded due to our criteria) who tested for ANCA at a single center in 10 years was considered, and life status and comorbidities of subjects were collected. We compared the overall survival of ANCA-positive and ANCA-negative patients by means of Kaplan-Meier curves, while a multivariable adjusted Cox regression was used to evaluate the association between the ANCA status and the outcome (death) in terms of hazard ratios (HR) with 95% confidence intervals (CI).

**Results:**

The positivity of perinuclear ANCA (pANCA) increased significantly mortality (HR, 1.60; 95% CI, 1.10–2.32), while cytoplasmic ANCA (cANCA) positivity failed to show a significant association (HR, 1.43; 95% CI, 0.77–2.68). The increased mortality rate was observed for both pANCA and cANCA in patients suffering from rheumatic disorders. No association was found between mortality and anti-MPO (HR, 0.63; 95% CI, 0.20–2.00) or anti-PR3 (HR, 0.98; 95% CI, 0.24–3.96) after adjusting for confounders.

**Conclusions:**

Serum pANCA and cANCA are independent negative prognostic factors in patients with concurrent autoimmune diseases.

## Introduction

Antineutrophil cytoplasmic antibodies (ANCA) are autoantibodies primarily involved in the pathogenesis of a subset of small-vessel vasculitides [ANCA-associated vasculitides (AAV)]. AAV include granulomatosis with polyangiitis (GPA, previously called Wegener granulomatosis), microscopic polyangiitis (MPA), and eosinophilic granulomatosis with polyangiitis (EGPA, previously Churg-Strauss syndrome) (1, 2). ANCA are detected by indirect immunofluorescence (IIF) and chemiluminescence immune assay (CLIA). By means of IIF, two main patterns can be observed: cytoplasmic (cANCA) and perinuclear (pANCA).

The presence of ANCA is not specific to AAV only (3–6). In a large observational study conducted on rheumatological patients and published in 2001, presence of ANCA (mainly pANCA) was also confirmed in a minor proportion of patients with other disorders, such as rheumatoid arthritis (RA) and systemic lupus erythematosus (SLE), along with ulcerative colitis, autoimmune hepatitis, and sclerosing cholangitis (3). In 2010, a different study, which was carried out on a large cohort of unselected patients, reported positive ANCA testing also among those with infections, malignancies, and other disorders (4). These results were not unexpected, as previous smaller studies and clinical reports had anticipated the presence of ANCA in a wide variety of diseases (5).

By means of chemiluminescence immunoassay (CLIA), it is possible to detect antigen specificity of ANCA. ANCA can target several antigens, but the main target antigens of ANCA associated with AAV are myeloperoxidase (MPO) and proteinase 3 (PR3) (7).

As far as the pathogenic role of ANCA is concerned, MPO has been reliably shown to induce vasculitis in mice through different study designs (8, 9). On the contrary, proof of PR3 pathogenicity has long been elusive to researchers working with animal models (10). This may be due to the different conformation of proteinase 3 between mice and humans, with human PR3 containing a hydrophobic patch which is absent in its murine counterpart (11, 12). Furthermore, PR3-ANCA vasculitis seems to be associated with a particular HLA allele (HLA-DPB1*04) (13).

More than three decades after their initial appearance, ANCA are still a fundamental adjunct to clinical presentation in the diagnostic and classification workflow of small-vessel vasculitis (7, 14). In EGPA, the presence of pANCA is strongly associated with the phenotype of the disorder and the organs affected (15). The presence of “natural,” apparently nonpathogenic, ANCA was also proven in some studies (16, 17). In these studies, ANCA were observed in the serum of healthy people. As a result, it seems that ANCA pathogenicity is bound to other conditions, possibly specific HLA alleles or immune dysregulation. It is also well conceivable that not all ANCA are equal, with some having higher titer and antigen affinity than others.

Whatever the case, it is currently unclear what the prognostic meaning of ANCA in patients with miscellanous disorders is, if any. Based on their proposed pathogenicity, we hypothesized that ANCA are associated with a worse prognosis irrespective of the presence of AAV. Thus, we investigated the association between the presence of serum ANCA and the overall survival in a population of Italian patients tested at a single tertiary centre, in a 10-year interval.

## Methods

This study was designed as a retrospective cohort study, where exposure was represented by ANCA positivity, and the outcome was patient’s death.

### Study Population

Sera from 6,285 patients were tested for the presence of ANCA in the Istituto Clinico Humanitas (ICH) laboratory in Rozzano, Italy. Testing was ordered by immunologists, rheumatologists, gastroenterologists, neurologists, and other physicians. All samples were first tested by IIF and, when positive, by CLIA, for anti-MPO and anti-PR3 (ANCA Test System, Immunoconcept; RA-MPO and RA-PR3 ZENIT, Menarini Diagnostics, Florence, Italy).

**Figure 1:**
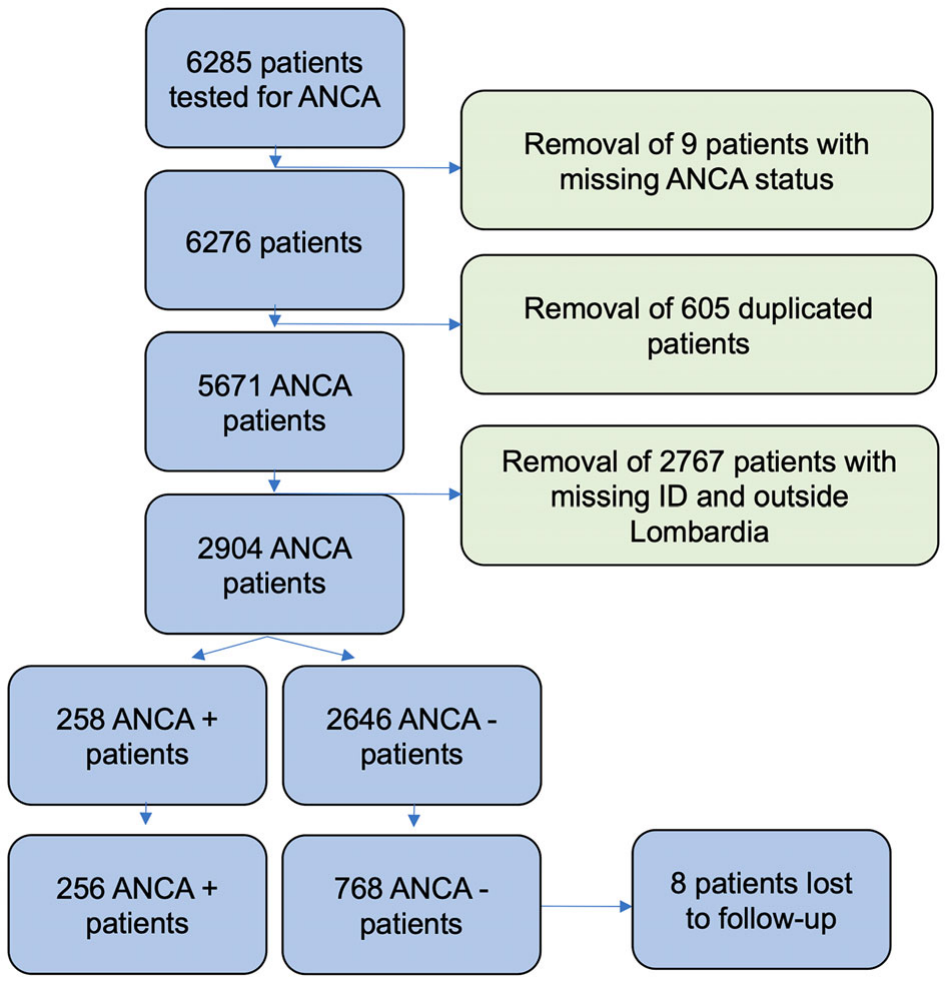
Flowchart of study selection process.

After applying eligibility criteria (**Supplemental Methods**), 2,904 patients where included in our study (**Figure 1**). In order to reduce the chance of selection bias, both ANCA-negative and ANCA-positive patients data were retrieved from the same database of a tertiary hospital (Istituto Clinico Humanitas). Furthermore, the unexposed population (ANCA negative) was matched with the exposed population (ANCA positive) for potential confounders. The 258 patients with ANCA were considered exposed and matched in a 1:3 ratio with 774 patients without the factor of exposure through a propensity score-based algorithm [RStudio, package MatchIt (18)] (**Table S1**) . Eight patients were lost to follow-up (two exposed and six unexposed patients). Potential confounders were identified as age, gender, and the “time of blood draw”, which is the number of days which elapsed between a predetermined date (January 1, 2006) and the date on which the blood sample was drawn for ANCA testing. Accounting for the date on which patients were tested (“time of blood draw”) was deemed necessary due to the extensive duration of our study and the significant variation in immunoassay accuracy and treatment strategies in the last two decades (19, 20). After adjustment for confounders, presence of different rheumatic diseases was considered an effect modifier. Data concerning presence of rheumatic disease and comorbidities were retrieved from the electronic health records of the hospital. While it is plausible that some data about comorbidities may be missing from our records (in case patients were treated by physicians external to the hospital), it was considered that this lack of information should affect patients independently from their ANCA status, and therefore it would not introduce a systematic bias into our study. Data concerning status of life were retrieved from the Italian Health registry system. The Humanitas institutional review board approved the present retrospective study.

### Statistical Analysis

In this retrospective cohort study, data originated from a period of 10 years, between November 9, 2007 and November 9, 2017. The follow-up was calculated from the day on which the blood sample was drawn for ANCA testing to the time of death or, if this was not observed, to November 9, 2017. Data were collected in October 2018.

A preliminary statistical analysis was carried out by obtaining the overall survival by means of Kaplan-Meier curves among exposed and unexposed patients. Log-rank test was performed to assess the statistical significance of the difference between the two groups. Multivariable-adjusted Cox regression was used to evaluate the association between the exposure and the outcomes of interest in terms of hazard ratios (HR).

We identified *a priori* age, gender, and the day on which the blood sample was drawn as potential confounders. The proportionality of hazards was confirmed with the Schoenfields residuals. As matching was performed only based upon the general ANCA status and not the presence of specific ANCA subtypes, possible confounding variables (age, gender, day of blood sample being drawn) have been added as covariates in the Cox regression stratified for specific ANCA. Linearity of continuous variables was checked by comparing models with the linear term to the model with restricted cubic splines. Potential effect modifications were also included and tested by likelihood ratio test.

Furthermore, to assess the potential effect modification by the presence of rheumatic disease, the effect of ANCA on survival was tested on an additive and multiplicative scale (21). Interaction on an additive scale can be calculated using the relative excess risk due to interaction (RERI), and means that the combined effect of two exposures is larger (or smaller) than the sum of the individual effects of the two exposures, whereas interaction on a multiplicative scale means that the combined effect is larger (or smaller) than the product of the individual effects (21).

With a power of 0.8, a sample size of 1,024 patients and an alpha error of 0.05, our study was able to detect a HR no smaller than 1.19. Statistical analyses were performed with Stata 15 and RStudio.

## Results

Before matching, a total of 2,904 entries were included. The majority of patients were women (59.3%), and the mean age was 58.78 years (SD, 17.24 years); of these, 258 patients were ANCA positive, accounting for a prevalence of 8.88%.

Before matching, the mean age of patients was not balanced (**Table S1**). The features of our matched population clearly reflected those of the exposed patients (ANCA+) (**Table S1**).

The main comorbidities of our matched population are depicted in **Table 1**. Among our patients, one-fourth (256) suffered from an immunological disorder.

**Table 1:** Comorbidities and ANCA status in the matched population.

	ANCA− (IIF)	cANCA+ (IIF)	pANCA+ (IIF)	Anti-PR3+ (ELISA)	Anti-MPO+ (ELISA)	Sum*
Age (year ± SD)	64.0 ± 16.9	65.9 ± 18.0	63.4 ± 18.7	60.6 ± 19.8	66.0 ± 15.4	
Male %	36.8	31.0	39.4	41.1	50.5	
Time of blood draw (days)	2,702 ± 1,1	2,305 ± 1,1	2,796 ± 1,1	3,046 ± 8	2,777 ± 8,1	
mean ± SD	24	79	00	98	71	
GPA (No. (%))	4 (0.5)	6 (10.3)	3 (1.5)	4 (23.5)	2 (6.5)	12 (1.2)
EGPA (No. (%))	3 (0.4)	–	6 (3.0)	–	4 (12.9)	9 (0.9)
MPA (No. (%))	–	–	2 (1.0)	–	2 (6.5)	2 (0.2)
UC (No. (%))	4 (0.5)	4 (6.9)	18 (8.9)	3 (17.6)	–	25 (2.4)
PSC (No. (%))	–	–	1 (0.5)	–	–	1 (0.1)
RA (No. (%))	19 (2.5)	1 (1.7)	10 (4.9)	–	–	30 (2.9)
SLE (No. (%))	4 (0.5)	1 (1.7)	2 (1.0)	1 (5.9)	–	6 (0.6)
PNS (No. (%))	18 (2.3)	1 (1.7)	5 (2.5)	–	2 (6.5)	24 (2.3)
Other (No. (%))	554 (72.1)	28 (48.2)	93 (45.8)	6 (35.3)	8 (25.8)	673 (65.7)
Other RD (No. (%))	95 (12.4)	9 (15.5)	32 (15.8)	1 (5.9)	2 (6.5)	136 (13.3)
CD (No. (%))	2 (0.3)	–	3 (1.5)	–	–	5 (0.5)
IHD (No. (%))	33 (4.3)	4 (6.9)	8 (3.9)	2 (11.8)	–	45 (4.4)
CVD (No. (%))	12 (1.6)	–	3 (1.5)	–	–	15 (1.5)
CNS (No. (%))	9 (1.2)	1 (1.7)	1 (0.5)	–	–	11 (1.0)
RLV (No. (%))	–	1 (1.7)	12 (5.9)	–	10 (32.2)	13 (1.3)
AIH (No. (%))	4 (0.5)	1 (1.7)	3 (1.5)	–	1 (3.2)	8 (0.8)
PBC (No. (%))	7 (0.9)	1 (1.7)	1 (0.5)	–	–	9 (0.9)
Sum*	768	58	203	17	31	1,024

GPA, granulomatosis with polyangitis; EGPA, eosinophilic granulomatosis with polyangitis; MPA, microscopic polyangitis; UC, ulcerative colitis; PSC, primary sclerosing cholangitis; RA, rheumatoid arthritis; SLE, systemic lupus erythematosus; PNS, neuropathies; Other, all disorders not included in different categories; Other RD, all the rheumatic disorders not included in different categories; CD, Crohn’s disease; IHD, ischemic heart disease; CVD, nonischemic cardiovascular disease; CNS, central nervous system disorders; RLV, renal-limited vasculitis; AIH, autoimmune hepatitis; PBC, primary sclerosing cholangitis.

*Sum of pANCA and cANCA is higher than 256 (261) due to the presence of patients with ANCA double positivities.

The mean follow-up duration was 3.9 years (unexposed 3.97; exposed 3.78). The total time at risk was 4,018 person-years (unexposed 3,049, exposed 968). A total of 157 deaths were observed. Eight patients (six without ANCA and two with ANCA) were lost to follow-up (missing status of life in Health registry system of Lombardia).

**Figure 2:**
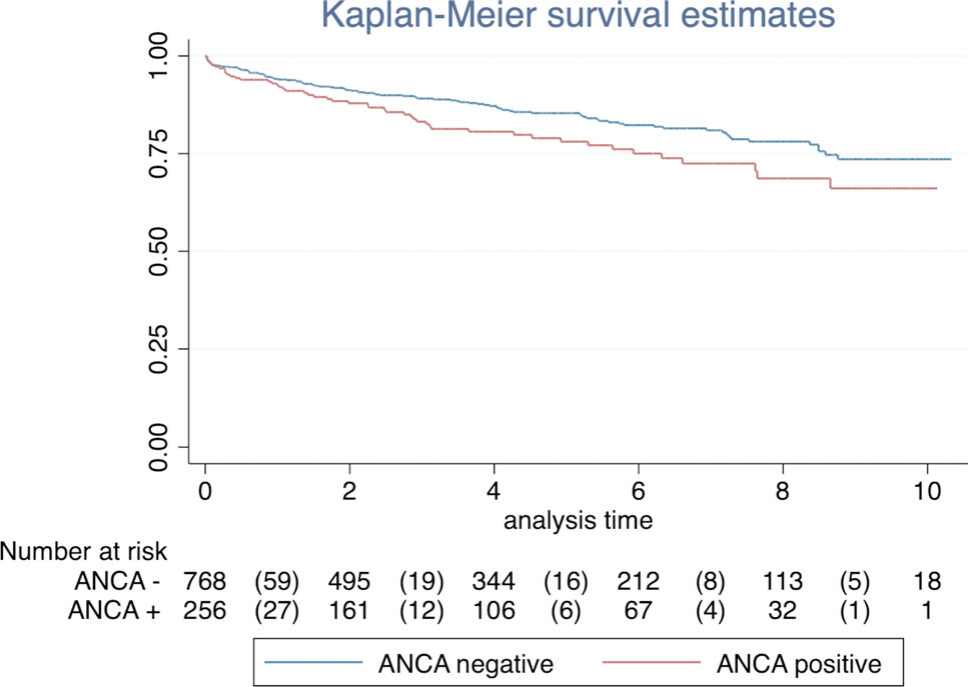
The association of ANCA status with overall survival. The Kaplan-Meier curve showed an overall 10-year event-free survival of 0.74 ± 0.03 (95% CI, 0.67 to 0.79) in ANCA-negative group and 0.66 ± 0.05 (95% CI, 0.55 to 0.75) in ANCA-positive group (log-ranks test *p* < 0.029).

The presence of ANCA, after matching adjustment, significantly increased the mortality rate (HR, 1.45; 95% CI, 1.04–2.03). Kaplan-Meier curves stratified by the exposure are reported in **Figure 2**. There was evidence of difference in the overall survival between ANCA and non-ANCA groups (Log-rank test for equality of survivor functions with *p* = 0.029).

The effect of the ANCA status on survival was then evaluated by stratifying patients with cANCA, pANCA, or no ANCA at all (**Table 2**). After adjusting for confounders, the presence of cANCA did not increase the mortality rate (HR, 1.43; 95% CI, 0.77–2.68). On the contrary, the presence of pANCA increased the mortality rate (HR, 1.60; 95% CI, 1.10–2.32). No association was found between mortality and anti-MPO (HR, 0.63; 95% CI, 0.20–2.00) or anti-PR3 (HR, 0.98; 95% CI, 0.24–3.96) after adjusting for confounders.

**Table 2:** Crude and adjusted estimate effect of ANCA status on mortality rate.

	HR	[95% CI]	Adjusted HR	[95% CI]
**No ANCA**	1.00	–	1.00	–
**cANCA**	1.27	0.68–2.37	1.43	0.77–2.68
**pANCA**	1.51	1.05–2.18	1.60	1.10–2.31

Adjustment for age, sex, and time of blood draw.

Based on the initial findings, it was hypothesized that the presence of autoimmune disorders may modify the effect of ANCA on mortality. The interaction of all autoimmune disorders (including IBD, RA, PSC, PBC, AIH, AAV, and others) with pANCA and cANCA was therefore considered. The effect modification of ANCA by rheumatic disease is reported in **Table 3**. The presence of cANCA and pANCA patients suffering from rheumatic disease was associated with higher mortality compared with reference population (respectively, HR, 3.12; 95% CI, 1.28–7.55 and HR, 2.08; 95% CI, 1.08–4.01). Patients without ANCA in absence of rheumatic disease have been considered reference category (stratum with the lowest risk of death).

**Table 3:** Effect modification of ANCA by rheumatic disorders on mortality rate.

	ANCA absent		cANCA		pANCA		HRs (95% CI) for ANCA within strata of rheumatic disorders	
	*N* deaths/alive	HR (CI 95%)	*N* deaths/alive	HR (CI 95%)	*N* deaths/alive	HR (CI 95%)	cANCA	pANCA
Rheumatic disorders absent	90/536	1	4/28	0.75 (0.27–2.03); *p* = 0.565	20/90	1.31 (0.80–2.15); *p* = 0.272	0.75 (0.27–2.05); *p* = 0.577	1.23 (0.75–2.02); *p* = 0.406
Rheumatic disorders present	17/125	0.98 (0.58–1.65); *p* = 0.930	7/14	3.12 (1.28–7.55); *p* = 0.012	19/74	2. 08 (1.08–4.01); *p* = 0.028	2.54 (1.01–6.37); *p* = 0.047	2.00 (1.03–3.89); *p* = 0.040

HRs are adjusted for age, sex, and time of blood draw.

Furthermore, to obtain all the information needed to assess effect modification of rheumatic disease (13), HRs for ANCA status within strata of immunological disorders are reported in **Table 3**. Among patients without rheumatic disease, there was no evidence of an effect on mortality of cANCA and pANCA compared with no ANCA (respectively, HR, 0.75; 95% CI, 0.27–2.05 and HR, 1.23; 95% CI, 0.75–2.02). Among patients with rheumatic disease, the mortality rate was more than double compared with patients without ANCA. The RERI was 2.39 (95% CI, −0.14–6.93) and 0.79 (95% CI, −0.86–3.20) for cANCA and pANCA, respectively. On multiplicative scale, the effect modification was 3.387 and 1.626, respectively. To verify that the effect modification of ANCA by rheumatic disease was not restricted to vasculitides only, a sensitivity analysis was carried out excluding patients affected by AAV (**Table 4**). Results were consistent with the previous analysis confirming that the effect modification observed was not ascribable to AAV.

**Table 4:** Effect modification of ANCA by rheumatic disorders excluding patients affected by ANCA-related vasculitides on mortality rate.

	ANCA absent		cANCA		pANCA		HRs (95% CI) for ANCA within strata of Rheumatic disorders	
	*N* deaths/alive	HR (CI 95%)	*N* deaths/alive	HR (CI 95%)	*N* deaths/alive	HR (CI 95%)	cANCA	pANCA
Rheumatic disorders absent	90/536	1	4/28	0.74 (0.27–2.03); *p* = 0.565	20/90	1.32 (0.80–2.12); *p* = 0.30	0.75 (0.27–2.05); *p* = 0.577	1.23 (0.75–2.02); *p* = 0.406
Rheumatic disorders present	14/121	0.87 (0.49–1.54); *p* = 0.634	3/13	4.08 (1.15–14.43); *p* = 0.029	15/55	2.82 (1.36–5.85); *p* = 0.006	2.55 (1.01–6.37); *p* = 0.047	2.00 (1.03–3.89); *p* = 0.040

HRs are adjusted for age, sex, and time of blood draw.

Using the same sequence of analysis, the effect modification of ANCA by rheumatoid arthritis and AAV are reported in **Tables S2, S3**, respectively. Because of low sample size in some strata, we could just focus on these two disorders. For the same reason, we could only assess pANCA effect in rheumatoid arthritis analysis.

Patients with pANCA (**Table S2**) and rheumatoid arthritis had higher mortality compared with patients without ANCA without rheumatoid arthritis (HR, 9.55; 95% CI, 1.85–49.5). Among patients without rheumatoid arthritis, there was no evidence of an effect of pANCA compared with no ANCA (HR, 1.38; 95% CI, 0.94–2.04). On the contrary, among patients with rheumatoid arthritis, the mortality rate was higher in patients with pANCA compared with patients without ANCA (HR, 10.80; 95% CI, 1.64–71.2). The RERI was 8.68, while on multiplicative scale, the effect modification was 7.804.

Analysis in AAV confirmed that there was no positive effect modification of the presence of ANCA across strata of AAV on an additive scale (**Table S3**).

## Discussion

Our retrospectively analyzed data from 1,024 Italian patients tested for the presence of serum ANCA indicate that the prevalence of ANCA affects the survival of tested subjects in a fashion depending on the IIF pattern and the rheumatological comorbidities.

First and foremost, we observed that the ANCA prevalence is similar to the one reported from other countries. Existing differences among populations are likely to originate from the heterogeneity of indications for testing and patient’s selection, although true geographical variations in the prevalence of ANCA may also exist. A comparison of our unmatched population with similar studies performed in the past (summarized in **Table S4**) demonstrates a strong heterogeneity. Still, several trends emerge from the evaluation. First, the prevalence of ANCA positivity seems to be lowest in the general population, it is greater in patients with nonspecific indications, and is highest among rheumatological patients (4, 17, 22–25). This finding is also confirmed by the different prevalence of ANCA among patients with (44.5%) and without (18.5%, *p* < 0.00001) autoimmune diseases within the whole cohort of patients. Second, in our study, the number of patients actually diagnosed with AAV is a small fraction of the tested subjects, but the percentage increases in those patients with a specific rheumatological indication for ANCA testing (3). In the present study, the low yield of the test for detection of AAV was probably due to different indications. There is a single study which reported both age and gender of patients with ANCA. It found results similar to ours (4). The prevalence of ANCA in both cohorts was higher among females and the elderly than it would be expected from the overall composition of the population.

An increase in overall mortality was observed in all patients with ANCA with respect to matched controls and the presence of ANCA, already adjusted with matching, increased the mortality rate, while this effect on mortality rate was restricted to pANCA after matching. When the presence of a rheumatic disease was accounted as a potential effect modifier, we observed that cANCA and pANCA increased mortality only in the presence of rheumatic diseases. These findings were confirmed by a sensitivity analysis carried out when we excluded patients with AAV, confirming that the effect modification was not ascribable to AAV. Furthermore, when AAVs were tested as a potential effect modifier, we were unable to confirm a positive effect modification induced by the presence of ANCA across strata of AAV on RERI and multiplicative scale.

We believe that these results have a potentially remarkable clinical impact since they suggest that ANCA define a subset of immunological patients with a more aggressive course of disease while the mechanisms can only be hypothesized. On one hand, it is possible that ANCA are the consequence of a more extensive systemic immune dysfunction. A plausible mechanism is an increased formation of ANCA in patients with higher production of NETs. NETs, which are implicated in the development of ANCA, are also involved in promoting the inflammatory state in RA, SLE, and other autoimmune disorders (26–29). ANCA could hence be just an indicator of a longstanding break in immune tolerance with increased NET production and associated systemic inflammation. On the other hand, ANCA may be directly pathogenic in a specific context. As no effect was observed in patients without rheumatic diseases, it is likely that their pathogenicity is dependent on different factors. ANCA may elicit damage only under conditions that are present in autoimmune diseases, namely, alterations in the activation of complement and cellular immunity (30, 31). In this setting, ANCA may favor neutrophils recruitment to sites of inflammation (32). Furthermore, NET production may also be stimulated by ANCA in predisposed patients, with a resulting increase in disease aggressiveness (28). Clearly, the two hypotheses are not exclusive if a circular model of inflammation is considered. ANCA may be at the same time an epiphenomenon of a more aggressive course of disease and a further mediator of inflammatory activity.

Our results are in partial agreement with a study conducted by Drooger et al. in 2009 (33) although differences in the size of the populations and enrolment criteria make comparison difficult. These authors investigated 54 patients with ANCA and a control group matched in 1:1 ratio. In addition, the presence of an AAV or other disorder was not considered an effect modifier. These features prevent us from understanding whether the observed increase in mortality in their study was due to the association of ANCA with AAV. In spite of a relatively long follow-up of 4.3 years, no events (deaths) were observed in their control group, thus raising the possibility of a selection bias. Finally, it should be considered that the primary aim of that study was the comparison of patients with anti-PR3 and anti-MPO, with the first group eventually found to have a decreased survival than the second (45% *vs.* 81%).

In our study, data about the effects of ANCA in AAV may have been incomplete due to the limitations of testing. Anti-LAMP2 are not commonly tested in these patients and false negatives (due to ceruloplasmin fragments) may also have occurred (34, 35).

Moreover, our results may have been limited by not accounting for patients’ comorbidities during matching or during the subsequent statistical analysis. Due to the retrospective nature of our study, we were unable to define which comorbidities were already present before patients entered the study. We assumed that both ANCA-negative and ANCA-positive patients did not significantly differ in their baseline status for factors other than their ANCA status. If this assumption proved to be wrong, the validity of our results would be doubtful.

Another limitation of our study is the discrepancy of results between ANCA testing with indirect immunofluorescence (highly associated with mortality) and testing with immunoassays (ELISA, not associated with mortality). This may be due to several reasons. Possibly, earlier ELISA tests were not as sensitive as IIF, and this reduced accuracy was reflected in our results (19, 36). However, this effect should be absent from future studies, as more recent immunoassays (ELISA, CLIA) have been proven to be as accurate as (or more accurate than) IIF (37). It is also plausible that the effect we observed on mortality is mediated by ANCA-targeting antigens other than MPO and PR3.

Of note, the striking increase in mortality risk observed in RA patients with pANCA has been observed for the first time in our study. As it applies to only five patients affected by RA and with pANCA positivity, this finding should be considered with extreme caution. It is however consistent with the results of a study published in 1997, in which the presence of ANCA (especially pANCA) was strongly associated with nephropathy and a more aggressive course of disease in RA (38). Another study published in 2000 also found ANCA to be correlated with the presence of rheumatoid factor and erosive bone disease in RA (39). These findings were not confirmed by other studies that found no association between the severity of RA and the presence of ANCA (40, 41).

Recently, a case-control study was published in which the presence of serum ANCA several years before the onset of ANCA-associated vasculitis was observed (42). While this study clearly applies to a different setting, it promotes the pathogenic nature of ANCA in humans.

In conclusion, this is the first study to investigate mortality associated with ANCA outside the setting of vasculitis, and its preliminary results should prompt future research efforts to provide more evidence on this issue, either to support or confute our findings.

## Data Availability Statement

The datasets presented in this article are not readily available because data requests will be evaluated and consent given only within the boundaries of the Italian regulations. Requests to access the datasets should be directed to enrico.brunetta@humanitas.it.

## Ethics Statement

The studies involving human participants were reviewed and approved by Istituto Clinico Humanitas IRCCS Rozzano, Italy. The ethics committee waived the requirement of written informed consent for participation.

## Author Contributions

EnB and GR designed the study and wrote the first draft. ElB and EV helped with data acquisition and study review. EM contributed with data analysis. MF, MDS, RF, CA, and CS contributed to writing, organizing, and reviewing the final manuscript. CS contributed to coordinating the effort of the individual researchers. All authors contributed to the article and approved the submitted version.

## Conflict of Interest

The authors declare that the research was conducted in the absence of any commercial or financial relationships that could be construed as a potential conflict of interest.

## Publisher’s Note

All claims expressed in this article are solely those of the authors and do not necessarily represent those of their affiliated organizations, or those of the publisher, the editors and the reviewers. Any product that may be evaluated in this article, or claim that may be made by its manufacturer, is not guaranteed or endorsed by the publisher.

## Abbreviations

ANCA, antineutrophil cytoplasmic antibodies; AAV, ANCA-associated vasculitides; GPA, granulomatosis with polyangiitis; MPA, microscopic polyangiitis; EGPA, eosinophilic granulomatosis with polyangiitis; IIF, indirect immunofluorescence; CLIA, chemilumiscence immune assay; ELISA, enzyme-linked immunosorbent assay; MPO, myeloperoxidase; PR3, proteinase 3; RA, rheumatoid arthritis; SLE, systemic lupus erythematosus; IBD, inflammatory bowel diseases; PSC, primary sclerosing cholangitis; PBC, primary biliary cirrhosis; AIH, autoimmune hepatitis; NET, neutrophil extracellular trap; LAMP2, lysosome-associated membrane protein 2; HR, hazard ratios; SD, standard deviation; RERI, relative excess risk due to the interaction.
