# Supplementary material for: Serum ANCA and Overall Mortality: A 10-Year Retrospective Cohort Study on 1,024 Italian Subjects
Source: Front Immunol. 2021 Sep 10;12:714174. doi: 10.3389/fimmu.2021.714174 (PMC8461098; doi:10.3389/fimmu.2021.714174)
Supplement: Supplementary file 1 [file DataSheet_1.docx]

**Supplemental Methods**

Eligibility criteria

- All patients who had more than one ANCA testing were only counted once.
- If all tests were negative, the earliest negative test was kept. If all tests were positive, the earliest positive test was kept. If results were mixed, the earliest positive test was kept.
- All patients with missing ID were removed.
- All patients who were not resident in Lombardia region were removed. This was due to difficulties in retrieving data about their status of life.
- Patients who had low-titer pANCA at IIF, with high-titer ANA and negative MPO/PR3 ELISA, were considered to be false positives.

**Supplemental tables**

|  | Before matching | | | After matching | | |
| --- | --- | --- | --- | --- | --- | --- |
|  | ANCA negative | ANCA positive | p | ANCA negative | ANCA positive | p |
| n | 2646 | 258 | - | 768 | 256 | - |
| Age (mean (SD)) | 58.26(17.07) | 64.15(18.03) | <0.001 | 64.02 (16.94) | 64.30 (18.04) | 0.821 |
| Gender = Male (%) | 1087 (41.1) | 96 (37.2) | 0.254 | 283 (36.8) | 95 (37.1) | 1.000 |
| Time of blood draw (mean (SD))  from 1^st^ jan 2006 | 1972.75 (1129.70) | 2109.55 (1140.08) | 0.064 | 2701.66 (1124.03) | 2673.86 (1141.58) | 0.733 |

**Table E1**. Before matching mean age is significantly different between the two groups. After performing the matching, age gender and time of blood draw were equally distributed among exposed and unexposed patients. Data of patients lost to follow-up are not included.

|  | ANCA absent | | pANCA | | HRs (95% CI) for ANCA within strata of arthritis |
| --- | --- | --- | --- | --- | --- |
|  | **N deaths/alives** | **HR**  **(CI 95%)** | **N deaths/alives** | **HR**  **(CI 95%)** | **pANCA** |
| rheumatoid arthritis absent | 105/644 | 1 | 34/159 | 1.38  (0.94-2.05); p= 0.1 | 1.38  (0.94-2.04; p=0.101 |
| rheumatoid arthritis present | 2/17 | 0.48  (0.12-1.96);  p= 0.310 | 5/5 | 9.55  (1.85-49.50); p=0.007 | 10.80  (1.64-71.2); p=0.013 |

**Table E2.** Effect modification of pANCA by rheumatoid arthritis on mortality rate. HRs are adjusted for age, sex, time of blood draw.

|  | ANCA absent | | cANCA | | pANCA | | HRs (95% CI) for ANCA within strata of AAV | |
| --- | --- | --- | --- | --- | --- | --- | --- | --- |
|  | **N deaths/alives** | **HR**  **(CI 95%)** | **N deaths/alives** | **HR**  **(CI 95%)** | **N deaths/alives** | **HR**  **(CI 95%)** | **cANCA** | **pANCA** |
| AAV absent | 104/657 | 1 | 7/40 | 1.13  (0.53-2.46); p=0.743 | 35/145 | 1.67  (1.13-2.47);  p= 0.009 | 1.13  (0.52-2.45); p=0.749 | 1.66  (1.13-2.46); p=0.010 |
| AAV present | 3/4 | 2.30  (0.72-7.37); p= 0.158 | 4/2 | 1.21  (0.275.48); p=0.805 | 4/19 | 0.55  (0.12-2.53); p=0.450 | 0.43  (0.07-2.67); p=0.367 | 0.49  (0.08-3.02); p=0.442 |

**Table E3.** Effect modification of ANCA by vasculitides on mortality rate. HRs are adjusted for age, sex, time of blood draw.

|  | Stone et al., 2000^49^ | McLaren et al., 2001^50^ | Schoenermarck et al., 2001^55^ | Tsiveriotis et al., 2011^52^ | Deshpande et al., 2016^53^ | Chehroudi et al., 2018^54^ | Our patients |
| --- | --- | --- | --- | --- | --- | --- | --- |
| Number of patients (ANCA + by IIF) | 856 (102) | 2734 | 4620 (624) | 10803 (661) | 198 (10) | 1889 (240) | 2904 (258) |
| Prevalence of ANCA by IIF | 11.9% | 18% in rheumatological patients, lower for other indications | 13.5% | 6% | 5.1% | 12.7% | 8.88% |
| Age (ANCA+) | - | - | - | 52.1 (60.1) | 50 | - | 58.78 (64.15) |
| Females (ANCA+) | - | - | - | 58.7% (62%) | 48.5% | - | 59.26% (62,7%) |
| AAV patients (ANCA+) | 69 (46) | - | 470 (359) | 121 (113) | - | 40 (40) | 36 (29) |
| Country | United States | United Kingdom | Germany | Greece | Australia | Canada | Italy |
| Cohort features | All wards | All wards | Only rheumatology | All wards | General population | All wards | All wards |

**Table E4**. Comparison of our population with previous studies
